# Enhancement of Immunoregulatory Function of Modified Bone Marrow Mesenchymal Stem Cells by Targeting SOCS1

**DOI:** 10.1155/2018/3530647

**Published:** 2018-05-08

**Authors:** Xiaoming Zhang, Fei Hua, Ziying Yang, Yueqiu Chen, Xiaomei Teng, Haoyue Huang, Yunfeng Zhao, Zhenya Shen

**Affiliations:** ^1^Department of Cardiothoracic Surgery, Suzhou Municipal Hospital, Suzhou, China; ^2^Department of Cardiovascular Surgery of The First Affiliated Hospital & Institute for Cardiovascular Science, Soochow University, Suzhou, China

## Abstract

**Objective:**

The study aim to investigate the role of microRNA-155 (miR-155) on the immunoregulatory function of bone marrow mesenchymal stem cells (MSCs).

**Methods:**

MSCs were isolated from 2-week-old Sprague-Dawley rats and identified by flow cytometry using anti-CD29, anti-CD44, anti-CD34, and anti-CD45 antibodies. MSCs were transfected with miR155-mimics, miR155-inhibitor, and control oligos, respectively, and then cocultured with spleen mononuclear cells (SMCs). The mRNA levels of Th1, Th2, Th17, and Treg cell-specific transcription factors (Tbx21, Gata3, Rorc, and Foxp3, resp.) and the miR-155 target gene SOCS1 were detected by quantitative real-time PCR (qPCR) in SMCs. The proportion of CD4^+^ FOXP3^+^ Treg cells was detected by flow cytometry. In addition, the effects of MSCs transfected with miR-155 on the migration of rat SMCs were investigated by transwell chamber.

**Results:**

CD29 and CD44 were expressed in MSCs, while CD34 and CD45 were negative. The percentage of CD4^+^ FOXP3^+^ Treg cells in the SMC population was significantly higher compared with that noted in SMCs control group (*p* < 0.001) following 72 hours of coculture with miR155-mimics-transfected SMCs. In contrast, the percentage of CD4^+^ FOXP3^+^ Treg cells in the SMCs cocultured with miR155-inhibitor-transfected MSCs was significantly lower compared with that noted in SMCs control group (*p* < 0.001). MiR155-mimics-transfected MSCs inhibited the expression of* Tbx21*,* Rorc,* and* SOCS1*, while the expression of* Gata3* and* Foxp3* was increased. In contrast to the downregulation of the aforementioned genes, miR155-inhibitor-transfected MSCs resulted in upregulation of* Tbx21*,* Rorc*, and* SOCS1* expression levels and inhibition of* Gata3* and* Foxp3*. In the transwell assay, miR155-mimics-transfected MSCs exhibited lower levels of SMCs migration, while the miR155-inhibitor-transfected MSCs demonstrated significantly higher levels of migration, compared with the blank control group (*p* < 0.01, resp.).

**Conclusion:**

miR-155 favors the differentiation of T cells into Th2 and Treg cells in MSCs, while it inhibits the differentiation to Th1 and Th17 cells.

## 1. Introduction

Mesenchymal stem cells (MSCs) are multipotent stem cells which can be isolated from various sources including bone marrow, spleen, heart, and umbilical cord blood tissues [1, 2]. MSCs have been considered as a promising treatment for a majority of autoimmune and inflammatory diseases as well as transplant rejection cases due to their immune-regulatory functions. In the peripheral blood, MSCs can promote the survival and phagocytosis of neutrophils [3] and enhance the phagocytosis of monocytes [4]. MSCs further regulate B-cell functions via soluble factors and cell–cell contact* in vitro* and* in vivo*, resulting in the B-lymphocyte G0/G1 phase arrest and in the inhibition of plasma-cell differentiation [5].

During coculture of human MSCs with purified subpopulations of immune cells, MSCs alter the cytokine secretion profile of dendritic (DCs), T helper 1 (Th1), Th2, and natural killer (NK) cells in order to induce a potent anti-inflammatory phenotype. MSCs have been shown to decrease tumor necrosis factor alpha (TNF-alpha) secretion from mature DCs and interferon gamma (IFN-gamma) from Th1 cells, while they increase interleukin-10 (IL-10) secretion from DCs and IL-4 from Th2 cells [6]. A recent study confirmed that the administration of MSCs ameliorated experimental autoimmune uveoretinitis (EAU) in rats by decreasing the production of Th1 and Th17 cytokine levels, while concomitantly increasing Th2 and regulatory T cell (Treg) cytokines (IL-10 and transforming growth factor (TGF)-â) during the entire course of EAU [7]. The immunosuppressive properties of MSCs have been exploited in a number of experimental models of autoimmune diseases, such as Crohn's disease [8], multiple sclerosis (MS) [9], and rheumatoid arthritis [10], in which T cells and mainly Treg cells played a significant role. The aforementioned studies established that MSCs can regulate immune response. However, the molecular mechanism of their regulatory action with regard to different immune cells requires further investigation.

MicroRNAs (miRNAs) are small noncoding RNAs that posttranscriptionally regulate gene expression by targeting specific messenger RNAs (mRNAs) for degradation [11]. Recent evidence indicates that miRNAs play a key role in the regulation of immunological functions including innate and adaptive immune responses, development and differentiation of immune cells, and the prevention of autoimmunity [12]. miR-155 has been considered as an important regulator of the immune response by various studies. In dendritic cells [13] and macrophages [14], miR-155 has been implicated as a positive regulator of inflammatory cytokine production. A study has shown that despite the low levels of basal miR-155 expression in B cells, miR-155 was able to positively regulate antibody-mediated signaling [15]. miR-155 is also considered as a central modulator of T cell responses [16], based on evidence suggesting that miR-155 promoted the development of inflammatory T cells including the Th17 cell and Th1 cell subsets [17]. In contrast to these findings,* miR-155−/−* mice were highly resistant to experimental autoimmune encephalomyelitis (EAE) [17]. miR-155 may be further involved in the maintenance of the MSCs potent immunosuppressive capacity. In addition, miR-155 targets TAK1-binding protein 2 (TAB2) in MSCs in order to regulate iNOS expression and nitric oxide release, by which T cell proliferation and function were inhibited [18]. However, the role of miR-155 in the interaction between MSCs and the immune cells remains partially undiscovered.

The present study investigated the role of miR-155 in the immunosuppressive function of MSCs.

## 2. Methods and Materials

### 2.1. Animals

Sprague-Dawley (SD) rats were provided by the Laboratory Animal Center of Soochow University (Suzhou, China). Animals were maintained under specific pathogen-free and standard conditions. All experimental procedures involving animals were approved by the animal ethical committee of Soochow University.

### 2.2. Isolation of MSCs and SMCs

MSCs were isolated from rat bone marrow as previously described [19]. Briefly, bone marrow cells were isolated from femurs and tibias of SD rats aged between 10 and 14 days. Isolated cells were cultured in flasks with DMEM/F12 (Gibco, USA) supplemented with 10% fetal bovine serum (FBS, Gibco, USA) in a CO_2_ incubator at 37°C. Following 3 days of incubation, nonadherent cells were removed. Adherent cells were trypsinized and passaged at 80%–90% confluency. At passage number 3, the isolated cells were assessed with the use of conjugated antibodies for CD29, CD45, CD44, and CD34 (CD29-PE, CD45-PE, CD44-FITC, and CD34-FITC, BD Biosciences, USA) by flow cytometry [20]. At passage 3, osteogenic and adipogenic differentiation was assessed by measurement according to the manuscript of instructions.

SMCs were isolated from four-week-old healthy male SD rats that were anesthetized and sacrificed to extract the spleen. The spleen was cut into pieces and passed through a 100 *μ*m cell strainer to obtain single cell suspensions. The cell suspension was mixed with 1640 medium. The cell suspension was collected in a 50 ml centrifuge tube and centrifuged at 1,500 rpm for 5 min. The cell pellet was resuspended with 5 ml 1x red cell lysis buffer (Solarbio, China) and placed on ice for 2 to 3 min. A total of 30 ml PBS was then added and the cells were centrifuged at 1,500 rpm for an additional 5 min. Following 2 to 3 times of repeated washing with sterile PBS, the spleen mononuclear cell suspension was obtained by resuspending the cell pellet with RPMI 1640 complete medium. The cells were maintained at 37°C in a humidified atmosphere with 5% CO_2_.

### 2.3. Transfection of miRNA-155 Mimic and Inhibitor

The MSCs at passage number 4 were split and seeded in a 24-well plate at a density of 2 × 10^5^/well. 24 h following cell seeding, miR-155 mimic, mimics-nc, miR-155 inhibitor, and inhibitor-nc (Genepharma, Shanghai, China) were transfected in MSCs using Lipofectamine 2000 transfection reagent (Invitrogen, CA, USA) following the manufacturer's instructions. The cells were incubated at 37°C in the presence of 5% CO_2_ for 48 h and harvested with TRIzol reagent (Invitrogen) for RNA extraction. The expression of monocyte chemotactic protein (MCP-1) was detected by ELISA according to the instructions of ELISA kit.

### 2.4. Coincubation of MSCs with SMCs

The medium was removed and the SMCs were washed 2 to 3 times with PBS 24 h following transfection. The RPMI1640 medium containing 10% FBS was added. The rat spleen mononuclear cell suspension was prepared and the cell concentration was adjusted to 2 × 10^6^/ml. The cells were subsequently seeded to the 12-well plate and 100 *μ*l of IL-2 (100 U/ml) along with 5 *μ*l of CD3 (1 *μ*g/ml) was added. The flow cytometry analysis of isolated MSCs and CD4^+^ Foxp3^+^ Treg was carried out as described previously [19].

### 2.5. Real-Time qRT-PCR

The Tbx21, Gata3, Rorc, Foxp3, and SOCS1 mRNA levels of SMCs cocultured with or without MSCs were determined by qPCR using RevertAid First Strand cDNA Synthesis Kit (ThermoFisher, USA) and SYBR Green Realtime PCR Master Mix (ThermoFisher, USA) following the manufacturer's instructions. The primer sequences that were used for the amplification are listed in Table 1.

### 2.6. Transwell Migration Assay

MSCs (passage 4) were inoculated in 24-well plates at a density of 1 × 10^5^/well in 500 *μ*l of complete medium. miR-155 mimics and/or miR-155 inhibitor and control oligos were transfected in MSCs with lipofectamine 2000 24 h following seeding. The spleen mononuclear cells were adjusted to 1 × 10^5^ cells/ml in serum-free 1640 medium 24 h following seeding and 200 *μ*l of cell suspension was added to the upper transwell chamber. The transwell chambers were incubated at 37°C in the presence of 5% CO_2_ in a humidified incubator for 4 to 6 h. The nonmigrating cells in the upper chamber were scrubbed with a cotton swab and the membranes were carefully rinsed with PBS. The infiltrating cells in the chamber were fixed in methanol for 5 min, rinsed with PBS, stained with DAPI for 10 min, and rinsed again with PBS. The membranes were carefully cut and observed under an inverted fluorescence microscope. A total of 3 random high-power fields (200 times magnification) were selected for the quantification of cell number with the Image J software.

### 2.7. Statistical Analysis

Data are presented as mean ± SD and analyzed using ANOVA post hoc LSD test with SPSS18.0 (SPSS Inc., Chicago, IL, USA). The results are plotted using Prism 5 (GraphPad Inc., SanDiego, CA, USA). A *p* value lower than 0.05 (*p* < 0.05) was considered statistically significant.

## 3. Results

### 3.1. Characterization of Rat BM-MSCs and Coculture of BM-MSCs with Spleen Mononuclear Cells

**Figure 1:**
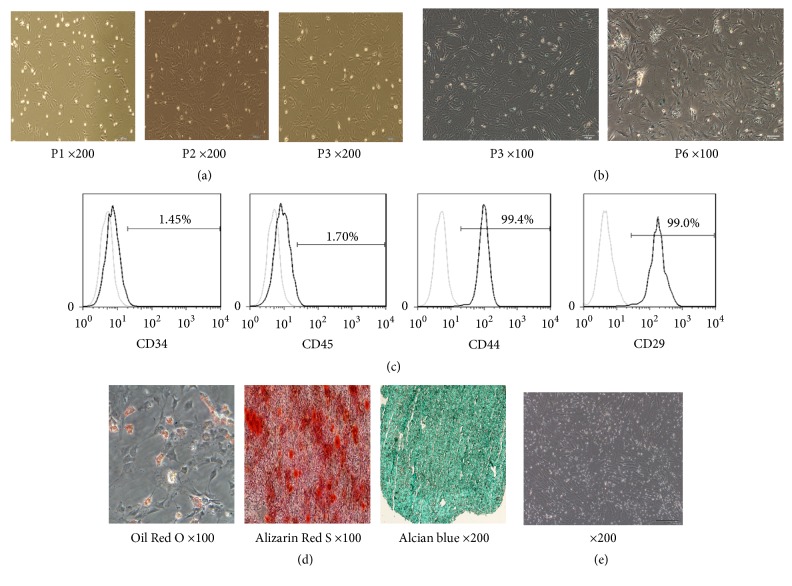
*Characteristics of rat BM-MSCs*. (a) Morphology of MSCs at passages 1, 2, and 3. (b) Beta-galactosidase staining of MSCs at passage numbers 3 and 6. (c) Cell surface makers of MSCs, assessed by flow cytometry at passage 3. (d) Differentiated MSCs strained by Oil Red O (left) or Alizarin Red S (right). Adipocyte differentiation was induced and cells were strained by Oil Red O. Bone differentiation of MSCs was induced and cells were strained by Alizarin Red S (200x). (e) Coculture of BM-MSCs and spleen mononuclear cells (SMCs). The attached strip-like cells are BM-MSCs, while the floating cells are SMCs. Original magnifications ×200 for (a), (b), and (d).

The cells exhibited spindle-shaped morphology following a few passages (Figure 1(a)). Following passage 6, the cell morphology was large and flat, and the proliferation rate was significantly decreased. The signs of senescence were observed (Figure 1(b)). The MSCs of passage numbers 3 to 5 were used for subsequent experiments.

It is well known that MSCs express certain surface markers, such as CD29, CD44, CD90, and CD105, whereas they lack the expression of the hematopoietic progenitor markers CD11b, CD14, CD34, or CD45 [21, 22]. In the present study, the expression of CD34, CD44, CD45, and CD90 was detected by flow cytometry. The results indicated that the isolated cells were CD44 and CD29 positive and CD34 and CD45 negative, indicating the isolation of pure MSCs that could be used for further experiments (Figure 1(c)). The differentiation adipogenic, osteogenic, and chondrogenic ability of MSCs is tested by Oil Red O, Alizarin Red S, and Alcian blue staining (Figure 1(d))

In order to simulate the inflammatory microenvironment, we cocultured SMCs with MSCs. The optimal ratio of spleen cells to the number of MSCs was maintained at 20 : 1. The CD3e antibody was added to the medium to stimulate the proliferation of SMCs and IL-2 was supplemented in order to maintain spleen mononuclear cell activity. In the coculture model, spleen mononuclear cells were suspended in the medium, and the adherent cells were MSCs (Figure 1(e)).

### 3.2. miR-155 Expression in MSCs

**Figure 2:**
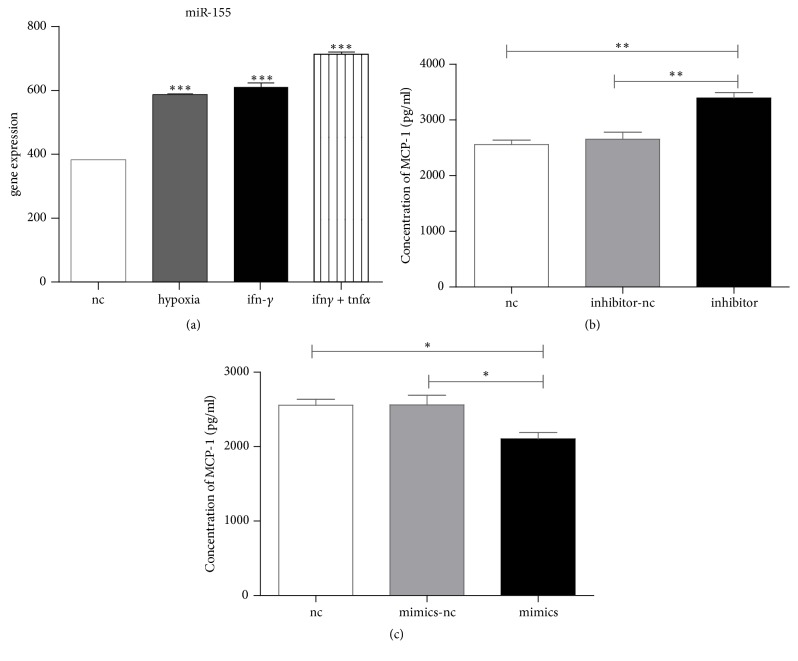
*Expression of miR-155 in BM-MSCs under different conditions*. The expression level was detected by QPCR. (a) Hypoxia and IFN*γ* or TNF*α* influence the expression of miR-155. (b) miR-155 level influence the expression of MCP-1 protein level. ^*∗*^*p* < 0.05, ^*∗∗*^*p* < 0.01, and ^*∗∗∗*^*p* < 0.001.

The results of the qPCR analysis demonstrated abundant expression of miR-155 in MSCs (Figure 2). During hypoxic treatment of MSCs in the presence and/or absence of IFN-*γ*, miR-155 levels were significantly increased (*p* < 0.001) (Figure 2(a)). Hypoxia and inflammatory factors including IFN-*γ* may affect the growth factor production and the activity of MSCs [23]. In this study, we have also shown that different miR-155 levels influence the expression of monocyte chemotactic protein (MCP-1) (Figure 2(b)). Consequently, it was expected that miR-155 may play a role in the immunosuppressive activities of MSCs.

### 3.3. The Effects of MSC miR-155 on the Expression of T Helper Cell-Specific Transcription Factors

**Figure 3:**
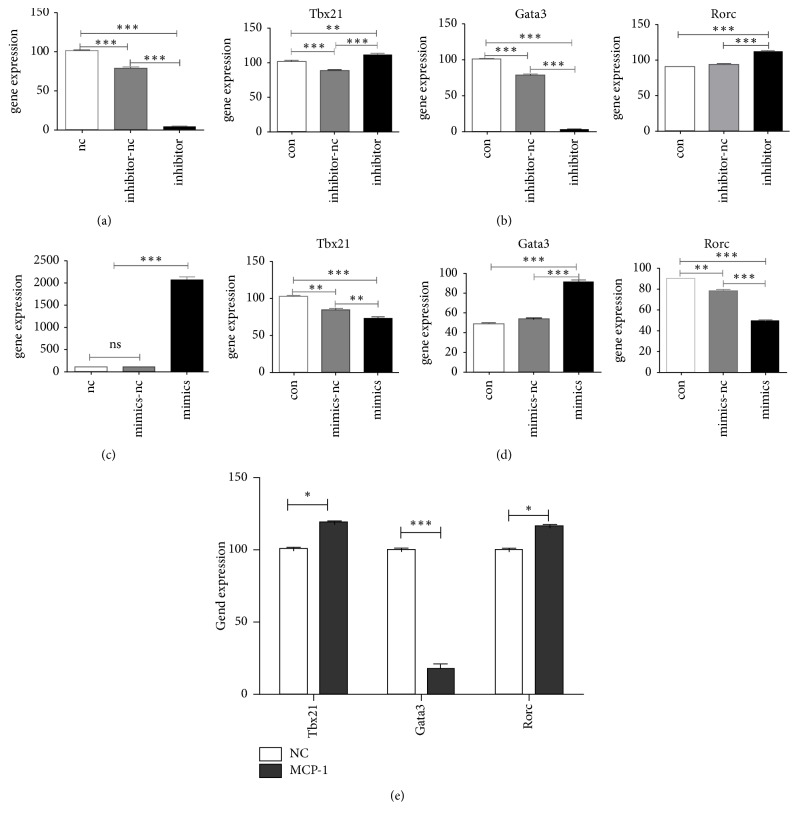
*Changes in BM-MSCs miR-155 levels affect T cell-specific transcription factors*. (a) miR-155 expression in BM-MSCs was blocked by inhibitor treatment. (b) Alterations of* Tbx21*,* Gata3,* and* Rorc* mRNA levels of SMCs following miR-155 inhibition in BM-MSCs. (c) Overexpression of miR-155 in BM-MSCs. (d) The effects of miR-155 overexpression on Tbx21, Gata3, and Rorc mRNA levels of SMCs. (e) The gene expression of Tbx21, Gata3, and Rorc under the influence of MCP-1. ^*∗*^*p* < 0.05, ^*∗∗*^*p* < 0.01, and ^*∗∗∗*^*p* < 0.001.

Since* Tbx21*,* Gata3,* and* Rorc* encode the specific transcription factors for Th1, Th2, and Th17 cells, respectively, the mRNA levels of these transcription factors were evaluated as indicators for T lymphocyte differentiation. The coculture of SMCs with miR155-overexpressing MSCs (Figure 3(a)) resulted in significantly lower mRNA levels of* Tbx21* and* Rorc* compared with SMCs control samples (*p* < 0.001 for both* Tbx21* and* Rorc*), whereas the* Gata3* level was higher (*p* < 0.001) (Figure 3(b)). In the SMCs and miR155-suppressed MSCs groups (Figure 3(c)), the expression levels of* Tbx21* and* Rorc* were significantly elevated (*p* < 0.01 for* Tbx21* and *p* < 0.001 for* Rorc*), while* Gata3* expression was downregulated (*p* < 0.001) (Figure 3(d)). As shown above, miR-155 influence the expression of MCP-1, so we detected the inflammation related gene Tbx21, Gata3, and Rorc expression (Figure 3(e)). The data indicated that miR-155 could promote the transformation of T cells to Th2 cells and inhibit the shift towards Th1 and Th17.

### 3.4. MSC miR-155 Promotes the Differentiation of Treg Cells

**Figure 4:**
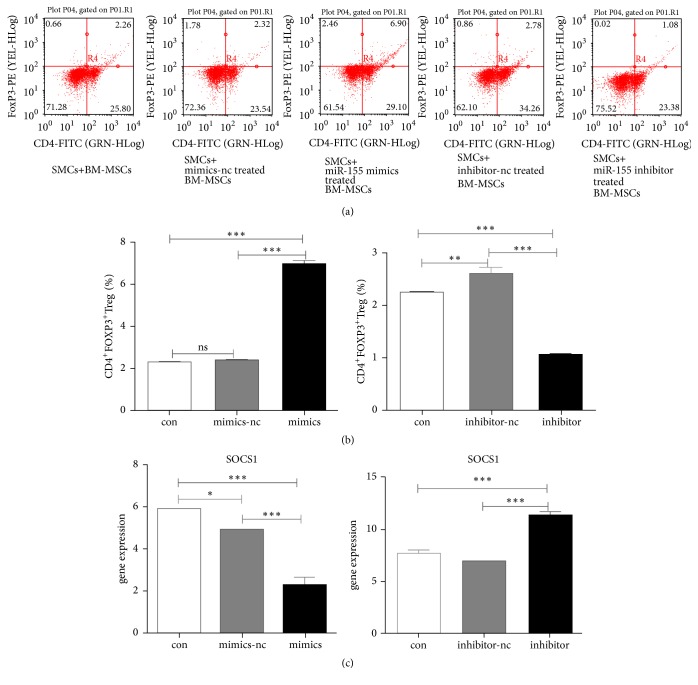
*Effect of BM-MSCs miR-155 levels on CD4* ^+^ *FOXP3* ^+^ *Treg cells*. (a) The proportions of CD4^+^FOXP3^+^Treg cells in SMCs incubated in the absence and/or presence of BM-MSCs. (b) Quantification of CD4^+^FOXP3^+^Treg cells in SMCs. (c) Overexpression and/or inhibition of miR-155 affected* SOCS1* mRNA levels in SMCs. ^*∗*^*p* < 0.05, ^*∗∗*^*p* < 0.01, and ^*∗∗∗*^*p* < 0.001.

Following 72 h of coculture, the percentage of CD4^+^ FOXP3^+^ Treg cells was detected by flow cytometry. The percentage of CD4^+^ FOXP3^+^ Treg cells was significantly higher in SMCs and miR155-mimics-transfected MSCs coculture groups compared with the SMSc control group (7.04 ± 0.15 versus 2.28 ± 0.02, *p* < 0.001) (Figure 4(b)). In contrast to these findings, the percentage of CD4^+^ FOXP3^+^ Treg cells was significantly lower in SMCs and miR155-inhibitor-transfected MSCs coculture groups compared with the SMSc control group (1.10 ± 0.02 versus 2.28 ± 0.02, *p* < 0.001) (Figures 4(a) and 4(b)).

Suppressor of cytokine signaling 1 (SOCS1) is highly expressed in Treg cells [24] and miR-155 has been shown to mediate SOCS1 repression that in turn contributes to Treg cell competitive fitness [25]. Therefore, the expression levels of* SOCS1* in SMCs were further investigated. The SMCs and miR155-mimics-transfected MSCs coculture groups exhibited significantly lower* SOCS1* mRNA levels, while the expression of* SOCS1 *was significantly elevated compared with the SMCs control group (*p* < 0.001) (Figure 4(c)). The findings indicated that the expression of miR-155 in MSCs may contribute to the differentiation of T cells into Treg cells.

### 3.5. MSC miR-155 Inhibits SMCs Migration

**Figure 5:**
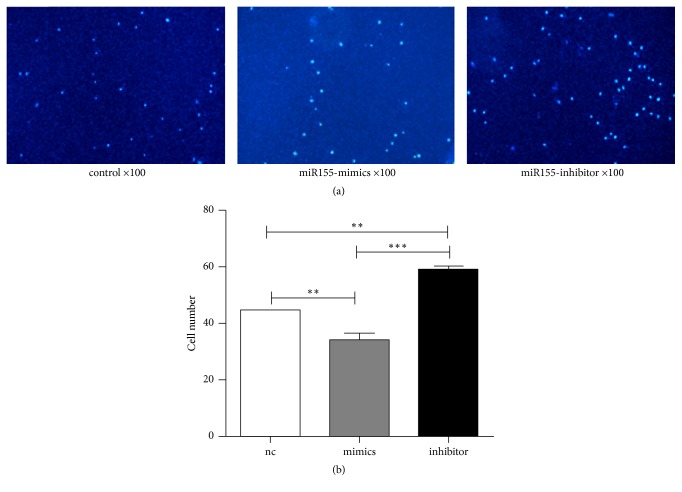
*BM-MSCs-derived-miR-155 affects the migration of SMCs*. (a) Infiltrating SMCs stained by DAPI were observed through OLYMPUS microscopy. (b) Quantification of SMCs migration counted by DAPI. ^*∗∗*^*p* < 0.01 and ^*∗∗∗*^*p* < 0.001.

The effects of MSC miR-155 on the recruitment of SMCs were investigated by the migratory capacity of SMCs in transwell cell assays. SMCs were loaded on the upper chamber and cultured in medium without serum, whereas the cells in the lower chamber were filled with serum containing medium (negative control, nc) and/or seeded with MSCs-transfected miR155-mimics and/or miR155-inhibitor. The number of spleen mononuclear cells in the miR155-mimics group was significantly lower than that in the negative control group (*p* < 0.01) (Figure 5). In contrast to this decrease in cell number, the number of spleen cells in the miR155-inhibitor group was significantly higher compared with that noted in the control group (*p* < 0.01) (Figure 5). The results suggested that high expression of miR-155 can inhibit the migration of SMCs.

## 4. Discussion

In the present study, miR-155 was reported to contribute to the immunosuppressive functions of MSCs by promoting the differentiation of T cells to anti-inflammatory Th2 and Tregs, while concomitantly inhibiting the differentiation to Th1 and Th17 subpopulations.

MSCs exhibit immunosuppressive effects by the direct modulation of T cell survival, proliferation, and differentiation [26]. However, MSCs can further modulate the behavior of T cells indirectly. Previous studies have shown that coincubation of dendritic cells (DCs) with MSCs could induce maturation of CD11c^low^CD45RB^+^ DCs and exert an immunosuppressive effect on T cells. T cells cocultured with CD45RB^+^ DCs expressed significantly higher levels of GATA-3 and Foxp3 and lower levels of T-bet and ROR*γ* compared with T cells cocultured with mature DCs [19]. The findings of the latter study are consistent with the data reported in the present study with regard to reduced* Tbx21* and* Rorc* and the increased* Gata3* and* SOCS1* mRNA level in MSCs that overexpress miR-155 (Figure 4). The production of DCs is stimulated in SMCs derived either from human or from rodent origin [27–29]. Therefore, it is possible that in our coculture model MSCs suppressed the proinflammatory role of T helper cells indirectly via DCs stimulation.

Monocyte chemoattractant protein-1 (MCP-1/CCL2) is known as one of the key chemokines that regulate migration and infiltration of monocytes/macrophages [30]. Moreover, MCP-1 favors the differentiation and migration of Th2 cells [31]. It has further been shown that the MSCs from patients with rheumatoid arthritis display impaired function with regard to the inhibition of Th17 cell polarization, which was related to the low expression of CCL2 in MSCs [32]. MCP-1 is highly produced by MSCs and acts on divergent intracellular signaling pathways [33]. Furthermore, inhibition of miR-155 upregulated the expression levels of MCP-1, IL-6, and IL-8 in bone marrow MSCs [34]. Therefore, we hypothesized that MCP-1 is one of the mediators of the immunosuppressive functions of MSCs. Based on the aforementioned studies it was expected that the mimics of miR-155 would inhibit the expression of MCP-1 in MSCs, while the miR-155 inhibitor would upregulate MCP-1 (Figure 2(b)). This may at least be partially explained by the effect of miR-155 transfected MSCs on the recruitment of immune cells (Figure 5). However, a comprehensive profiling of the downstream cytokines of miR-155 in MSCs is required in order to understand their effects on different cell populations in SMCs.

MicroRNAs regulate gene expression at a posttranscriptional level within the cells. However, recent evidence suggests that microRNAs can be transferred between cells in order to mediate target gene repression. It was reported that miR-155 and miR-146a were released from dendritic cells within exosomes and were subsequently internalized by recipient dendritic cells in order to reprogram the cellular response to endotoxin [35]. Furthermore, miR-155 and miR-146a are present in exosomes and pass between immune cells in mice [35]. In addition, miR-155 was identified in the exosomes from human bone marrow-derived MSCs [36] and was ranked as one of the top 20 miRNAs according to their expression levels in MSC-exosomes [37]. We further demonstrated that the production of exosomes was increased concomitantly with the upregulation of miR-155 following hypoxia and/or IFN-*γ* stimulation (data not shown). Therefore, it may be possible that MSCs regulate the expression levels of miR-155 target genes in T cells and other immune cells via exosome production.

The migratory ability of inflammatory cells is essential for the inflammatory cell infiltration and the number of infiltrating inflammatory cells can reflect the intensity of the inflammation. Leukocytes migrate to sites of tissue injury and infection in order to silence the inflammatory stimuli and contribute to tissue repair [38]. It was further reported that MSCs recruited various immune cells* in vitro* and in mouse models [39]. Therefore, the transwell migration assay was used in order to determine whether the miR-155 expression changes in MSCs affected the migration of immune cells. The results suggested that miR-155 could inhibit the migration of spleen mononuclear cells. Although a vast number of miR-155 target genes have been identified, notably in immune cells and various cancers (reviewed by Mashima [15]), the functions and downstream targets of miR-155 in MSCs are still unknown. In addition, the specific cell types(s) of migrating cells suppressed by MSCs have not been fully identified.

MSCs are ideal for cell-based therapy in various inflammatory diseases due to their immunosuppressive properties. However, various factors may influence their therapeutic effects, including administration routes, detection time-points, disease models, differentiation of MSCs* in vivo*, and timing and dosage of MSC administration. Recently, it was shown that the age of MSC donors affected the expression levels of miRNAs in mesenchymal stem cell-derived extracellular vesicles. For example, the expression levels of miR-146a, miR-155, and miR-132 decreased by 93 ± 3% with increasing donor age [40]. In the present study, we isolated MSCs from young rats and used low-passage cells for coculture experiments. Since miR-155 may be an important regulator of the immunosuppressive functions of MSCs, the age of animals and the passage of the MSCs should carefully considered in future studies.

In summary, the present study demonstrated that alterations of miR-155 levels in MSCs affected the differentiation of T cell subsets in SMCs, indicating that miR-155 may be important for the maintenance of the immunosuppressive functions of MSCs. Despite these findings, the identification of the targets of miR-155 in MSCs requires further investigation.

## Figures and Tables

**Table 1:** Primers used in qPCR.

Primers	Sequences (5′-3′)
GAPDH-F:	GTATCGGACGCCTGGTTACC
GAPDH-R:	ACCAGCTTCCCATTCTCAGC
Gata3-F:	ACCACCTATCCGCCCTATGT
Gata3-R:	CACAGTGGGGTAGAGGTTGC
Foxp3-F:	GGCACTTCTCCAGGACAGAC
Foxp3-R:	ACATTGATCCCAGGTGGCAG
Tbx21-F:	CCTCCATAAGTACCAGCCGC
Tbx21-R:	TTCTCCCGGAATCCTTTGGC
Rorc-F:	AAAACAGAGGTCCAAGGGGC
Rorc-R:	CTGTCTGAGCCCTGTTCTGG
SOCS1-F:	CCTTCGACTGCCTCTTCGAG
SOCS1-R:	CGGGTTAAGAGGGATGCGTG
